# Novel Adamantane–Sclareol Hybrids Exploit ROS Vulnerability to Overcome Multidrug-Resistance in Glioblastoma Cells

**DOI:** 10.3390/molecules30244756

**Published:** 2025-12-12

**Authors:** Ema Lupšić, Pavle Stojković, Marija Grozdanić, Nataša Terzić-Jovanović, Milica Pajović, Fani Koutsougianni, Dimitra Alexopoulou, Igor M. Opsenica, Milica Pešić, Ana Podolski-Renić

**Affiliations:** 1 Institute for Biological Research “Siniša Stanković”–National Institute of the Republic of Serbia, University of Belgrade, Despota Stefana 142, 11108 Belgrade, Serbia; ema.lupsic@ibiss.bg.ac.rs (E.L.); marija.grozdanic@ibiss.bg.ac.rs (M.G.); milica.pajovic@ibiss.bg.ac.rs (M.P.); 2 University of Belgrade–Faculty of Chemistry, P.O. Box 51, Studentski Trg 16, 11158 Belgrade, Serbia; pavle@chem.bg.ac.rs (P.S.); igorop@chem.bg.ac.rs (I.M.O.); 3 University of Belgrade–Institute of Chemistry, Technology, and Metallurgy, National Institute of the Republic of Serbia, Njegoševa 12, 11000 Belgrade, Serbia; nterzic@chem.bg.ac.rs; 4 Department of Pharmacology, Faculty of Medicine, School of Health Sciences, University of Thessaly, Panepistimiou 3 (Biopolis), 41500 Larissa, Greece; fkoutsoug@uth.gr (F.K.); dalexopoulou@uth.gr (D.A.)

**Keywords:** glioblastoma, multidrug resistance, collateral sensitivity, oxidative stress, adamantane, sclareol

## Abstract

Multidrug resistance (MDR) presents a significant challenge in the treatment of glioblastoma. We evaluated six novel adamantane–sclareol hybrids that integrate a natural labdane diterpene scaffold with an adamantane moiety to address this issue. Compounds **2**, **5**, and **6** demonstrated the ability to bypass P-glycoprotein (P-gp)-mediated resistance in resistant U87-TxR cells and induced collateral sensitivity, with compound **2** exhibiting the highest selectivity for glioblastoma compared to normal glial cells. Mechanistic studies revealed that compounds **2** and **5** selectively triggered early apoptosis in MDR cells, significantly elevated levels of H_2_O_2_ and peroxynitrite, and disrupted mitochondrial membrane potential. Additionally, these compounds altered the expression of key genes involved in glutathione (GSH) and thioredoxin (Trx) antioxidant defense systems and increased ASK1 protein levels, indicating the activation of ROS-driven apoptotic signaling. Both compounds inhibited P-gp function, leading to enhanced intracellular accumulation of rhodamine 123 (Rho 123) and synergistically sensitized U87-TxR cells to paclitaxel (PTX). A preliminary Rag1 xenograft study demonstrated that compound **5** effectively suppressed tumor growth without causing significant weight loss. Collectively, these findings position adamantane–sclareol hybrids, particularly compounds **2** and **5**, as promising strategies that exploit an MDR-associated reactive oxygen species (ROS) vulnerability, combining selective cytotoxicity, redox disruption, and P-gp modulation to eliminate resistant glioblastoma cells and enhance the efficacy of chemotherapeutics.

## 1. Introduction

Glioblastoma is the most common and aggressive form of brain tumor. It is characterized by genomic instability, uncontrolled cell proliferation, and invasiveness, traits that contribute to its aggressive nature [1]. The standard treatment protocol for glioblastoma is the Stupp protocol, which includes surgical resection followed by radiotherapy and concomitant temozolomide therapy [2]. However, the progression-free survival of glioblastoma patients is only 7 months, with a median overall survival of 14 months [3]. Despite the poor response to current treatment options, glioblastoma therapy has changed little over the past two decades due to limited alternatives, highlighting the urgent need for innovative therapies [4].

The existence of drug resistance is one of the main limitations to efficient glioblastoma treatment. Resistance to a large number of structurally and functionally different chemotherapeutics is referred to as multidrug resistance (MDR). One of the main mechanisms of MDR is increased expression of membrane transporter P-glycoprotein (P-gp), which belongs to the ATP-binding cassette (ABC) transporter family–ABCB1 [5]. On the membranes of cancer cells, P-gp is responsible for reduced accumulation of drugs within the cells, resulting in limited therapeutic efficacy [6]. P-gp is particularly important component of the blood–brain barrier, where it prevents the entry of toxic substances into the brain.

Dysregulated redox signaling has been directly linked to the overexpression of efflux transporters and MDR in cancer [7,8]. Under physiological conditions, reactive oxygen species (ROS) are important signaling molecules that are tightly regulated by the antioxidant defense system. Elevated ROS levels can disrupt essential cell functions, damage biomolecules such as DNA, proteins, and lipids, and ultimately trigger cell death [8]. Efflux transporters such as P-gp are upregulated in response to oxidative stress, allowing cells to facilitate drug efflux and maintain redox balance [9]. Glioblastoma cells adapt to the oxidative stress induced by chemotherapy by upregulating antioxidant systems such as glutathione (GSH) and thioredoxin (Trx), as well as enzymes such as superoxide dismutase (SOD) and catalase (CAT), thus promoting survival and contributing to therapy resistance [10]. GSH is an important antioxidant that neutralizes ROS either directly or as a cofactor for the enzymes GSH peroxidase (GPx) or GSH-S-transferase (GST). GPxs use GSH to reduce peroxides, forming oxidized GSH (GSSG), which is restored by GSH reductase (GR). GSTs detoxify xenobiotics by conjugating them with GSH [11]. CAT also decomposes hydrogen peroxide (H_2_O_2_) and overlaps in function with GPxs, while superoxide dismutases (SODs) convert superoxide radicals into oxygen (O_2_) and H_2_O_2_ [12]. The thioredoxin (Trx) system, consisting of Trx, Trx reductase (TrxR), and NADPH, maintains the cellular redox balance. Trx reduces various target proteins, while TrxR restores Trx using NADPH. One of the target proteins of Trx is apoptosis signal-regulating kinase 1 (ASK1). Reduced Trx inhibits ASK1 and prevents apoptosis, but under oxidative stress, reduced Trx levels decrease, leading to ASK1 activation and apoptotic signaling [13].

Collateral sensitivity refers to a phenomenon in which drug-resistant cancer cells are more sensitive to certain drugs compared to their drug-sensitive counterparts [11,14]. The term is usually applied to cells that overexpress P-gp. Several mechanisms have been proposed to explain collateral sensitivity. One important mechanism is the ability of drugs to alter the intracellular redox balance, leading to the formation of ROS [14]. These agents are able to induce ROS production in both MDR cells and their corresponding sensitive cancer cells, but MDR cancer cells often exhibit higher susceptibility to oxidative stress. This increased vulnerability may be due to altered metabolic activity, increased basal ROS levels, or impaired antioxidant defense systems commonly associated with the MDR phenotype [8,15,16]. Overall, collateral sensitivity represents a promising strategy to overcome MDR in cancer therapy by identifying MDR-selective agents and selectively eliminating resistant populations [14].

Natural products are a rich reservoir of bioactive substances. It is estimated that between 1981 and 2019, as many as 25% of approved anticancer drugs originated from natural sources [17]. Sclareol is a labdane-type bioactive diterpene isolated from the flowers of the plant *Salvia sclarea* L. (Clary Sage). In vitro studies have shown that sclareol exerts antiproliferative effects in breast, lung, colon and osteosarcoma cell lines, primarily through G0/G1 cell cycle arrest, the induction of apoptosis, and the modulation of membrane potential [18]. Notably, sclareol’s ability to cross the blood–brain barrier [19] further supports its therapeutic potential in glioblastoma treatment. Derivatization of sclareol has emerged as an important strategy to expand its bioactive framework and yielded a series of promising derivatives with good pharmacological profiles. In glioblastoma cells, sclareol–doxorubicin conjugates showed improved anticancer activity and selectivity over doxorubicin alone [20]. In addition, MDR glioblasto ma cells were collaterally sensitive to novel sclareol derivatives, which also showed a potential to inhibit P-gp [21].

Nanoparticles serve as effective carriers that enhance the delivery of anticancer agents by modifying pharmacokinetics, increasing intratumoral accumulation, and facilitating the controlled release of drugs [22]. Their application aims to reduce systemic toxicity, overcome drug resistance, and enable targeted treatment strategies. Our recent research has demonstrated that sclareol and other labdane diterpenes can spontaneously form nanoparticles without the need for external physical or chemical forces [23]. These nanoparticles exhibit uniform sizes and negative zeta potentials. Furthermore, sclareol derivatives, such as sclareol–doxorubicin hybrids and hybrids incorporating adamantane moieties, yield nanoparticles with positive zeta potentials, which have shown significant anticancer activity against glioblastoma and non-small cell lung carcinoma models [20,23].

**Figure 1:**
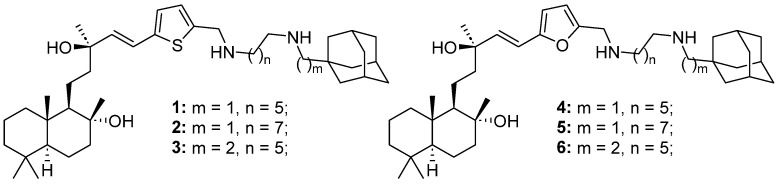
Structures of six adamantane–sclareol hybrid compounds.

In this study, we investigated six adamantane–sclareol hybrid compounds (Figure 1) whose synthesis was reported by Stojković et al. [23]. These hybrids showed nanoparticle characteristics, improved cytotoxicity, higher selectivity, and lower resistance profiles compared to sclareol in non-small cell lung carcinoma cells [23]. Herein, we used sensitive and MDR human glioblastoma cell lines, as well as a normal glial cell line, to assess their impact on cell death induction, mitochondrial membrane potential disruption, ROS production, and the GSH and Trx antioxidative defense systems. Additionally, we investigated their potential to sensitize MDR glioblastoma cells to paclitaxel (PTX) through modulation of P-gp activity.

## 2. Results

### 2.1. Novel Adamantane–Sclareol Hybrids Inhibit the Viability of Glioblastoma Cells

We assessed the viability of sensitive glioblastoma cell lines A-172 and U87, an MDR glioblastoma cell line U87-TxR, and a normal glial cell line SVG p12 after 72 h treatment with six adamantane–sclareol hybrids (**1**, **2**, **3**, **4**, **5**, **6**). Metabolic activity was assessed by 3-[4,5-dimethylthiazol-2-yl]-2,5-diphenyltetrazolium bromide (MTT) assay. All results are summarized in Table 1. The relative resistance factor represents the ratio of IC50 values determined between the MDR U87-TxR cell line and its sensitive counterpart U87. Compounds **2**, **5,** and **6** showed collateral sensitivity, with IC50 values at least 20% lower in the MDR glioblastoma cell line compared to its sensitive cell line (relative resistance factor < 0.8). Notably, **3** showed the highest selectivity toward glioblastoma cells over normal glial cells, with a selectivity index (SI) of 3.541, while **6** showed the lowest SI of 0.547. Although **6** showed collateral sensitivity, it lacked selectivity toward cancer cells. Compound **4** did not show collateral sensitivity or selectivity toward glioblastoma cell lines. In A-172 cells, the efficacy of most compounds was similar to that observed in U87 cells, except for **6,** whose effect was comparable to that in U87-TxR cells.

Our preliminary efficacy study of **5** in subcutaneous xenografts of U87 cells in Rag1 mice showed that 7 mg/kg, administered twice i.p., strongly inhibits tumor growth (Figure S1A). Tumors in the treated group remained significantly smaller than those in the control group over 12 days, with several time points showing significant differences. Excised tumors from the treated group are visibly smaller than those from the untreated group. Body weight is not adversely affected by **5** (Figure S1B). Treated animals start slightly lighter but gain weight over time and show no consistent significant weight loss. The maximal tolerated dose (MTD) for **5** was 25 mg/kg.

### 2.2. Cell Death Induction by Adamantane–Sclareol Hybrids Confirms Collateral Sensitivity

**Figure 2:**
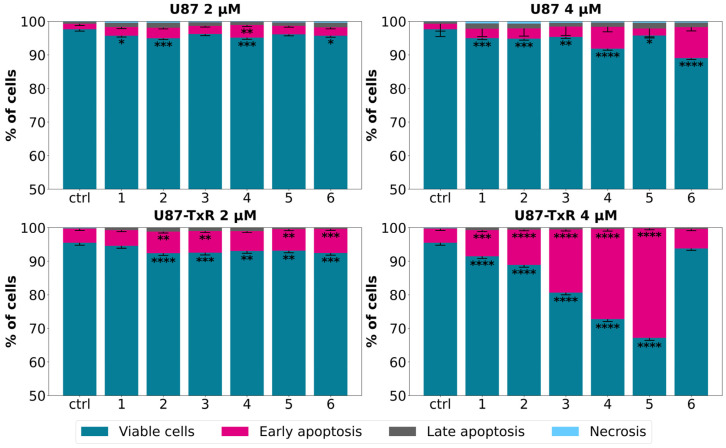
Cell death analysis in glioblastoma cells after treatment with adamantane–sclareol hybrids. Cell death was assessed with AV/PI staining in U87 and U87-TxR cells after 48 h treatment with compounds **1**, **2**, **3**, **4**, **5**, and **6** at concentrations of 2 µM and 4 µM. The results are presented with as histograms with percentages of viable (AV^−^ PI^−^), early apoptotic (AV^+^ PI^−^), late apoptotic (AV^+^ PI^+^), and necrotic (AV^−^ PI^+^) cells. At least 10,000 events were recorded per sample. Three separate experiments were performed (*n* = 3). Statistical analysis was executed using GraphPad Prism 8.0.2. via two-way ANOVA and Dunnett’s multiple comparisons test. A statistically significant difference between the treated samples and the untreated control is indicated as follows: * *p* ≤ 0.05, ** *p* ≤ 0.01, *** *p* ≤ 0.001, **** *p* ≤ 0.0001.

Following the MTT assay, we conducted further examination to determine whether the observed cytotoxicity was associated with cell death induction. The sensitive U87 cell line and its MDR counterpart U87-TxR were exposed to adamantane–sclareol hybrids at concentrations of 2 µM and 4 µM for 48 h, and then Annexin V–Propidium Iodide (AV/PI) staining was performed. Flow cytometry analysis showed that both concentrations induced only a modest increase in early and late apoptotic cells in U87 cells compared to untreated cells (Figure 2 and Figure S2). In contrast, treatment with compounds **2**, **3**, **4**, and **5** resulted in a significant increase in the percentage of early apoptotic cells, particularly at 4 µM in the MDR glioblastoma cell line (Figure 2). The early apoptotic population increased from 4.17% in untreated cells to 26.84% and 32.6% following treatment with **4** and **5**, respectively (Figure S3). Compounds **1**, **2**, **3**, **4**, and **5** demonstrated significantly better effects in MDR U87-TxR cells than in sensitive U87 cells at 4 µM, consistent with the MTT results. Collateral sensitivity was observed for all tested compounds at 2 µM (Figure 2). Based on their collateral sensitivity profile in MDR U87-TxR cells, confirmed by MTT and AV/PI assays, and their selectivity for glioblastoma cells, compounds **2** and **5** were chosen as suitable candidates for further evaluation.

### 2.3. Adamantane–Sclareol Hybrids Modulate Oxidative Stress and Decrease Mitochondrial Membrane Potential

**Figure 3:**
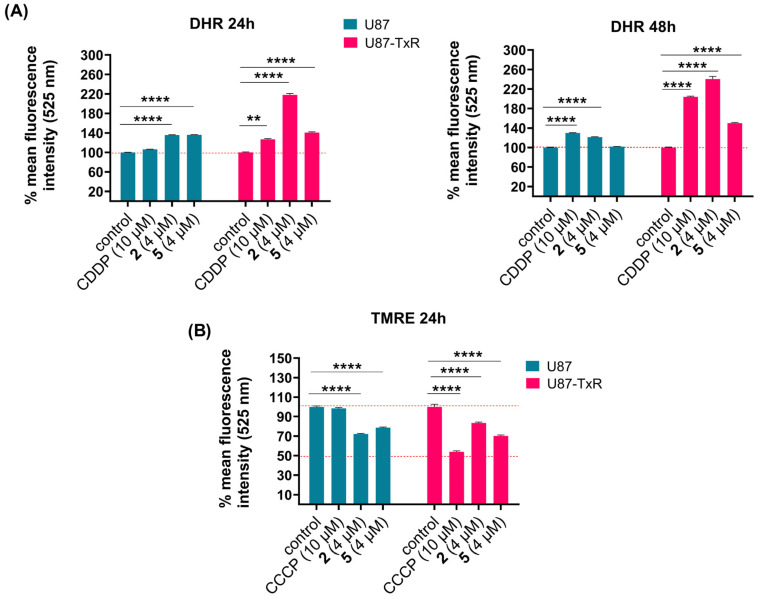
Changes in ROS production and mitochondrial membrane potential in U87 and U87-TxR cells following treatments with **2** and **5**. Changes in detection of ROS levels by DHR labeling (**A**) after 24 h treatment (**left**) and 48 h treatment (**right**) with **2** and **5** in U87 and U87-TxR cells. (**B**) Changes in mitochondrial membrane potential using TMRE staining after 24 h treatment with **2** and **5**. Results are presented as relative percentages of mean fluorescence intensity of untreated control set at 100%. Statistical analysis was performed using GraphPad Prism 8.0.2. with two-way ANOVA and Dunnett’s multiple comparisons test, comparing each treatment group to the untreated control group: ** *p* ≤ 0.01, and **** *p* ≤ 0.0001.

To assess whether compounds **2** and **5** affect ROS levels in U87 and U87-TxR cells, we measured the intracellular superoxide anion using dihydroethidium (DHE), peroxynitrite anion, and H_2_O_2_ using dihydrorhodamine 123 (DHR) via flow cytometry. The results obtained after 24 h and 48 h treatments are presented as the mean percentage of fluorescence intensity relative to the untreated control set at 100% (Figure 3A and Figure S4). Cisplatin (CDDP) was used as a positive control. DHE analysis showed no significant changes in intracellular superoxide anion levels (Figure S4). After DHR staining, U87 cells showed slight or no increase in H_2_O_2_ and peroxynitrite anion levels over the 24 h and 48 h treatments with both compounds (Figure 3A). However, changes were more evident in the MDR glioblastoma cell line. An increase in DHR fluorescence was observed after treatment with **2** and **5** at both time points (Figure 3A). The most pronounced increase in ROS levels was induced by **2** after 48 h treatment (Figure 3A). Since excessive ROS production can trigger mitochondrial dysfunction, we next assessed whether oxidative stress was accompanied by changes in mitochondrial membrane potential using tetramethylrhodamine ethyl ester (TMRE) staining, also via flow cytometry. Membrane potential was measured following 24 h treatment with 4 µM of **2** and **5** in U87 and U87-TxR cells. Carbonyl cyanide *m*-chlorophenyl hydrazine (CCCP), a known mitochondrial oxidative phosphorylation uncoupler, was used as a positive control. TMRE fluorescence decreased in both cell lines after treatment with **2** and **5**, indicating loss of mitochondrial membrane potential (Figure 3B). It is worth noting that both positive controls, CDDP and CCCP, achieved more potent effects in MDR U87-TxR cells than in sensitive U87 cells.

### 2.4. Differential Effects of Adamantane–Sclareol Hybrids on Oxidative-Stress-Related Gene Expression in Sensitive vs. MDR Glioblastoma Cells

**Figure 4:**
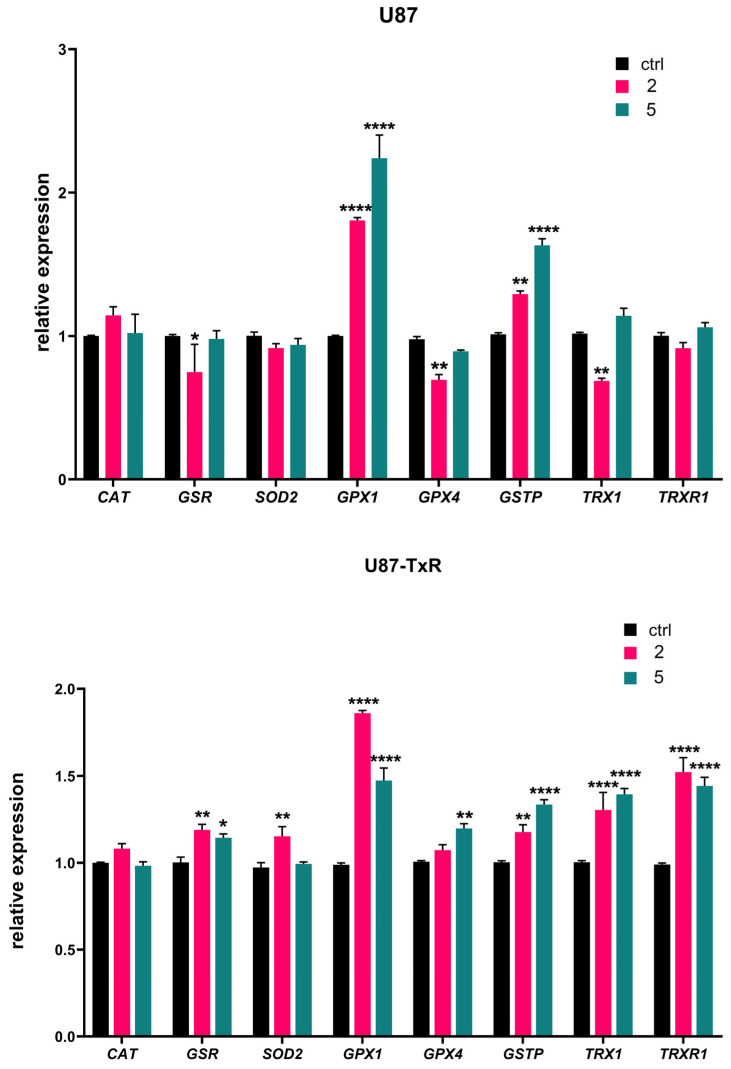
Changes in the mRNA expression of antioxidant-related genes induced by **2** and **5** in U87 and U87-TxR cell lines. Quantitative RT-PCR analysis of *CAT*, *GSR*, *SOD2*, *GPX1*, *GPX4*, *GSTP*, *TRX1*, and *TRXR1* mRNA expression in U87 and U87-TxR cells treated for 24 h with **2** and **5**. Expression levels were normalized to the housekeeping gene ACTB and are presented relative to untreated control cells set at 1. Data represent the mean ± SEM from three independent experiments. Statistical analysis was performed using two-way ANOVA followed by Sidak’s multiple comparisons test; statistically significant difference between the treated samples vs. control: * *p* ≤ 0.05, ** *p* ≤ 0.01, **** *p* ≤ 0.0001.

We further analyzed mRNA expression of key oxidative-stress-related genes (*CAT*, *GSR*, *SOD2*, *GPX1*, *GPX4*, *GSTP*, *TRX1*, and *TRXR1*) by RT-qPCR after 24 h treatment with adamantane–sclareol hybrids at a concentration of 4 µM (Figure 4). The highest increase in *GPX1* mRNA expression was observed upon treatment with **2** and **5** in U87 cells (1.8-fold and 2.2-fold, respectively) (Figure 4). Treatment with **2** and **5** also induced a pronounced increase in the expression of *GPX1* mRNA in U87-TxR cells (1.9-fold and 1.5-fold, respectively). As shown in Figure 4, both cell lines significantly increased *GSTP* gene expression following treatment with **2** and **5**. Components of the Trx system (*TRXR1* and *TRX1*) were upregulated in U87-TxR cells after treatment with adamantane–sclareol hybrids. In U87 cells, the mRNA levels of *TRXR1* and *TRX1* mainly remained at control levels. However, after treatment with **2**, *TRX1* mRNA showed significant downregulation, which was in contrast to the upregulation observed in U87-TxR cells (Figure 4). An increase in the mRNA expression of *SOD2*, *GSR*, and *GPX4* was observed only in the MDR glioblastoma cell line, while *CAT* mRNA expression levels remained unchanged upon treatment with **2** and **5** in both cell lines (Figure 4).

The quantitative RT-PCR results in Figure S5 reveal distinct changes in antioxidant transcripts in U87-TxR cells compared to the parental U87 cells. In U87-TxR cells, there is an increase in *CAT* and *GPX1*, demonstrating a heightened capacity for clearing H_2_O_2_. Conversely, a decrease in the expression of *GPX4*, *SOD2*, *GSR*, *GSTP*, *TRX1*, and *TRXR1* in U87-TxR was detected. Therefore, U87-TxR cells exhibit a shift towards improved soluble peroxide clearance but lose the ability to mitigate lipid peroxidation and have reduced mitochondrial and thiol-based antioxidant capacities.

### 2.5. Adamantane–Sclareol Hybrids Increase TrxR1 and ASK1 Protein Level in an MDR Glioblastoma Cell Line

**Figure 5:**
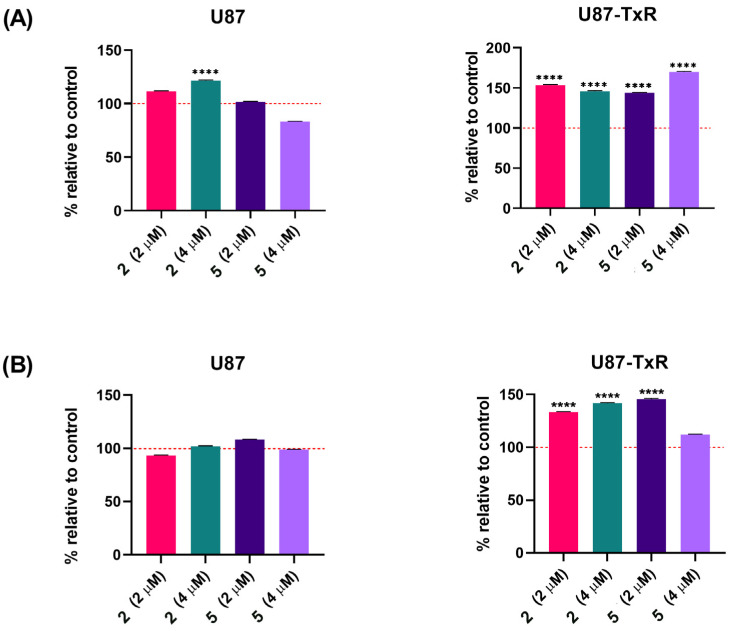
TrxR1 and ASK1 protein expression in U87 and U87-TxR cell lines following treatment with adamantane–sclareol hybrids. (**A**) TrxR1 and (**B**) ASK1 protein expression was assessed after 48 h treatment with **2** and **5** at concentrations of 2 µM and 4 µM. Data are presented as percentage relative to untreated control (100%, red dashed line), with statistical significance to the untreated control **** *p* < 0.0001. Statistical analysis was performed using one-way ANOVA followed by Dunnett’s post hoc test.

Next, we analyzed whether adamantane–sclareol hybrids affect the Trx target proteins TrxR1 and ASK1 in U87 and U87-TxR cells. Following 48 h treatment with **2** and **5** at concentrations of 2 µM and 4 µM, cells were immunostained with TrxR1 and ASK1 specific antibodies. All results are presented as a percentage change relative to the untreated control, set as 100% (Figure 5). In U87 cells, only treatment with 4 µM **2** caused a slight increase in TrxR1 levels (Figure 5A). The protein level of TrxR1 significantly increased in U87-TxR cells after treatment with both compounds. The most prominent increase in TrxR1 level (by 69%) was induced by 4 µM **5**. ASK1 protein levels increased in U87-TxR cells under the same treatment conditions (Figure 5B). However, ASK1 expression slightly increased after treatment with **5** at a 4 µM concentration, but this change was not statistically significant. In U87 cells, ASK1 level remained unchanged upon treatment with compounds **2** and **5**.

Protein analyses in Figure S6 show a significant decrease in Trxr1 levels between U87 and U87-TxR cells, which correlates with the *TRXR1* gene expression shown in Figure S5. The expression level of ASK1 remains unchanged in U87-TxR cells.

We further analyzed the potential of adamantane–sclareol hybrids to inhibit TrxR enzyme activity using a colorimetric assay for the detection of TrxR activity in cell lysates. This kinetic assay is based on the ability of TrxR to reduce its substrate 5,5ʹ-dithiobis(2-nitrobenzoic acid) (DTNB) to the yellow product 5-thio-2-nitrobenzoic acid (TNB) in the presence of NADPH. The absorbance of the produced TNB is considered equivalent to the enzymic activity of TrxR. In the kinetic assay, the absorbance of DTNB was measured over 20 min, resulting in a curve showing the dependence of absorbance on time. As expected, a positive control, aurothiomalate (ATM), inhibited TrxR activity, as evidenced by the lack of increase in absorbance over time. However, compounds **2** and **5** increased DTNB absorbance over time in a manner similar to DMSO, indicating that these compounds do not inhibit TrxR enzyme activity (Figure S7).

### 2.6. Adamantane–Sclareol Hybrids Inhibit P-gp Activity

**Figure 6:**
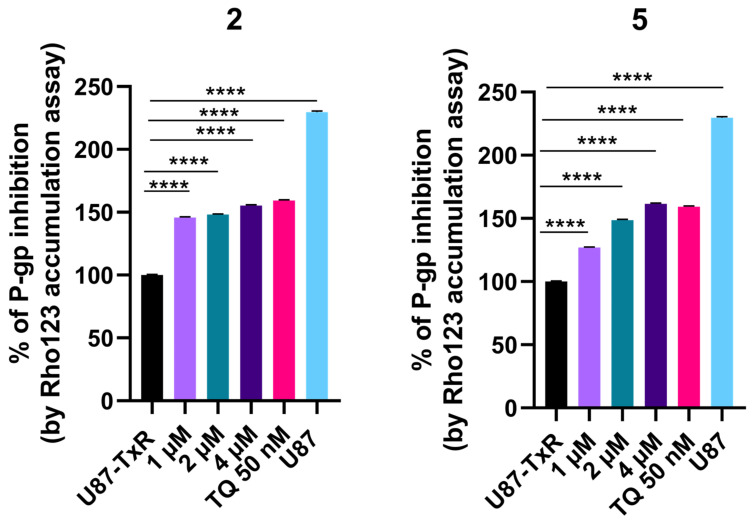
Concentration-dependent effect of **2** and **5** on P-gp activity. Percent of P-gp inhibition, expressed as an increase in Rho123 accumulation, relative to the Rho123 accumulation in U87-TxR cells set at 100%. TQ (50 nM) was used as a reference compound. Sensitive U87 cells are presented as positive control for Rho 123 accumulation. Statistical analyses were performed using one-way ANOVA followed by Dunnett’s multiple comparison test, **** *p* < 0.0001.

**Figure 7:**
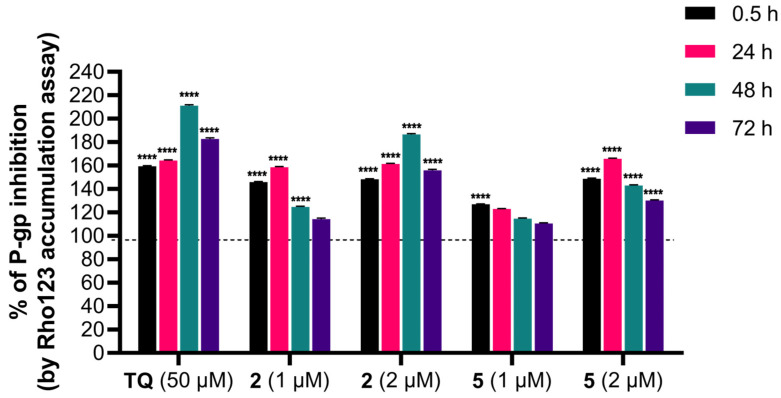
Time-dependent intracellular accumulation of Rho123 in MDR glioblastoma cells treated with **2** and **5**. Cells were treated with compounds **2** and **5** at concentrations 1 µM and 2 µM. TQ (50 nM) was used as a reference compound for P-gp inhibition. Intracellular Rho123 fluorescence was measured after 0.5 h, 24 h, 48 h, and 72 h of treatment. Data are presented as the percentage of P-gp inhibition, expressed as an increase in Rho123 accumulation, with 100% set according to the untreated U87-TxR cells (black dashed line). At least 10,000 events were recorded per sample. Data are expressed as mean ± SEM of three independent experiments (*n* = 3). Statistical analysis was performed using two-way ANOVA followed by Dunnett’s multiple comparison test, **** *p* < 0.0001.

The influence of adamantane–sclareol hybrids on P-gp activity was assessed by measuring intracellular accumulation of rhodamine 123 (Rho 123), a known P-gp substrate, in a P-gp-overexpressing MDR glioblastoma cell line. Tariquidar (TQ), a non-competitive P-gp inhibitor, was used as a reference compound. In the concentration-dependent assay, which lasted 30 min, both compounds increased Rho 123 accumulation, indicating their potential to modulate P-gp activity (Figure 6 and Figure S8). Both hybrids achieved accumulation comparable to TQ at the highest concentration (Figure 6 and Figure S8). We further assessed the time-dependent effect of compounds **2** and **5** on P-gp activity (Figure 7). Compound **2** enhanced the accumulation of Rho 123 at both concentrations applied within 24 h. This effect was maintained at 2 μM with prolonged exposure, reaching its maximum after 48 h. In MDR glioblastoma cells, treatment with 1 μM of **5** led to increased accumulation after 30 min, but this effect declined after 24 h, 48 h, and 72 h. In contrast, treatment with 2 μM of **5** resulted in the highest level of inhibition after 24 h. Although this inhibition remained significant over time, it decreased after 48 h and 72 h (Figure 7).

### 2.7. Adamantane–Sclareol Hybrids Sensitize MDR Glioblastoma Cells to PTX

**Figure 8:**
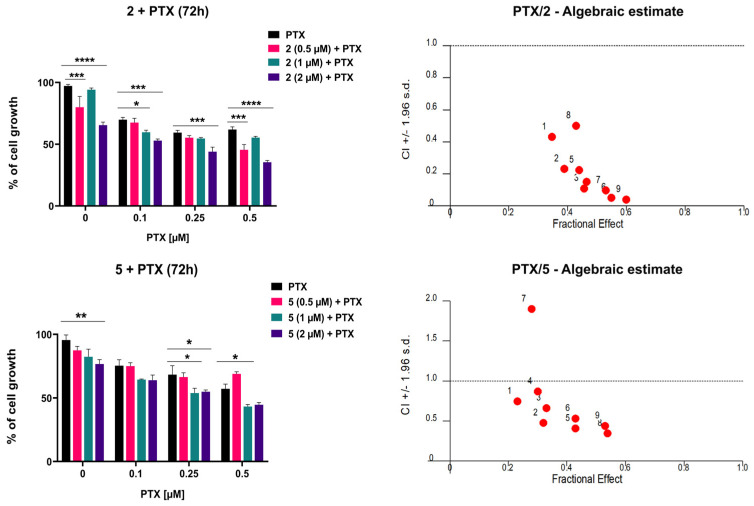
The combined effects of **2** and **5** with PTX in U87-TxR cells. Cells were treated for 72 h with increasing concentrations of PTX, alone and in combination with compound **2** or **5**. Cell viability was assessed by MTT assay. Results are presented as mean values ± SEM, obtained from three independent experiments (*n* = 3). Statistical analysis was performed by two-way ANOVA, comparing combination treatments to PTX alone. Statistical significance compared to untreated control cells is indicated as follows: * *p* ≤ 0.05, ** *p* ≤ 0.01, *** *p* ≤ 0.001, **** *p* ≤ 0.0001. The interactions between adamantane–sclareol hybrids and PTX were analyzed by CalcuSyn software. The results are shown in a fraction-affected CI graph. Values of CI < 1 point to a synergistic effect, CI values close to 1 indicate an additive effect, while CI > 1 indicates antagonism.

To evaluate whether compounds **2** and **5** potentiate the cytotoxic effect of paclitaxel (PTX) in MDR glioblastoma cells, their simultaneous combinations were assessed by MTT assays following 72 h treatment. Cells were exposed to three increasing concentrations of PTX (0.1 μM, 0.2 μM, and 0.5 μM), alone or in combination with three concentrations of **2** or **5** (0.5 μM, 1 μM, and 2 μM). Both compounds enhanced the inhibitory effect of PTX (Figure 8). Consistently, the IC50 values of PTX were significantly reduced in combination with **2** or **5** compared with PTX alone (Table 2). The most prominent decrease in IC50 was observed after treatment with 2 μM of compound **2**. These results were analyzed using CalcuSyn software for computerized synergism/antagonism analysis. All examined combinations of **2** and PTX demonstrated strong synergistic (CI < 1) interactions (Figure 8). In addition, combinations of **5** with PTX mainly exhibited synergistic (CI < 1) interactions (Figure 8). The sensitization of MDR glioblastoma cells is more pronounced with **2**, which clearly correlates with its higher potency in inhibiting P-gp and maintaining this effect over time.

## 3. Discussion

We evaluated six adamantane–sclareol hybrids in sensitive (U87, A-172) and MDR (U87-TxR) glioblastoma models and in a normal glial line (SVG p12) and selected compounds **2** and **5** for mechanistic follow-up based on their capability to induce collateral sensitivity and glioblastoma selectivity. Our key findings are that three hybrids (**2**, **5**, and **6**) induce collateral sensitivity in U87-TxR cells, while **2** displays the best cancer selectivity versus SVG p12.

At the structural and pharmacological levels, the present adamantane–sclareol hybrids build on earlier generations of sclareol-based derivatives that showed enhanced anticancer activity but more limited mechanistic insight into MDR reversal. Sclareol–doxorubicin conjugates improved antiproliferative activity and selectivity over doxorubicin alone in glioblastoma models [20]. However, they were primarily characterized as potent cytotoxic nanoparticles without a detailed evaluation of their impact on P-gp function or the redox vulnerabilities associated with MDR. Likewise, triazolopyrimidine–sclareol hybrids were reported to induce collateral sensitivity and to affect P-gp-overexpressing glioblastoma cells [21]. Still, their ability to functionally inhibit P-gp and remodel ROS-dependent signaling was not systematically assessed.

Hybrids **2** and **5** selectively induce early apoptosis in MDR U87-TxR cells, while both compounds increase H_2_O_2_/peroxynitrite levels, disrupt mitochondrial membrane potential, and alter the transcription of genes involved in the GSH and Trx redox systems in U87-TxR cells. Although TrxR1 and ASK1 protein levels are elevated in U87-TxR cells after treatment, TrxR enzymatic activity is not inhibited. Importantly, both hybrids inhibit P-gp activity and sensitize U87-TxR cells to PTX, with stronger and more sustained P-gp inhibition by **2** and more variable time-dependent inhibition by **5**. Our preliminary results with **5** on Rag1 xenografts are encouraging, but comprehensive toxicology, pharmacokinetics, and orthotopic glioblastoma models are necessary to evaluate the therapeutic window and brain delivery.

U87-TxR cells differ from U87 cells via altered antioxidant defenses. *GPX1* is increased, strengthening GSH-dependent reduction of H_2_O_2_, while *GPX4* is decreased, weakening detoxification of phospholipid hydroperoxides and increasing vulnerability to lipid peroxidation and ferroptosis [24]. Decreased *GPX4* expression is recognized as a key vulnerability of drug-tolerant persister cells [25]. *CAT* is increased, further supporting H_2_O_2_ clearance [26]. In contrast, *SOD2*, *GSTP*, *TRX1*, and *TRXR1* levels are reduced, indicating weaker mitochondrial superoxide dismutation by *SOD2* [27], less efficient glutathione recycling by *GSR* [28], diminished electrophile/xenobiotic conjugation by *GSTP* [29], and a reduced thioredoxin redox buffering (*TRX1*/*TRXR1*) [30]. Functionally, this profile favors soluble peroxide detox but compromises lipid ROS control and thiol redox resilience, creating a membrane-oriented ROS vulnerability.

In addition, differences in TrxR1 protein expression between U87 and U87-TxR cells are consistent with a remodeled Trx–ASK1 checkpoint [31]. Because TrxR1 keeps Trx levels low and restrains ASK1 activation, alterations in TrxR1 can lower the threshold for stress kinase signaling [31]. Therefore, U87-TxR cells possess asymmetric antioxidant strategy through enhanced H_2_O_2_ detox alongside weakened lipid-peroxide and Trx defenses paired with a sensitized stress-signaling axis. Therapies that elevate lipid ROS or perturb thiol redox control can exploit this gap [32], overtaking GPX1/CAT buffering and engaging ASK1-driven cell death pathways.

Mechanistic interpretation of our results points to changes in the redox balance. Namely, DHR-based measurements demonstrate that H_2_O_2_ and peroxynitrite increase preferentially in U87-TxR cells following **2** and **5** exposure, whereas superoxide detected by DHE is unchanged. The simultaneous loss of mitochondrial membrane potential supports a model in which peroxide accumulation and/or peroxynitrite formation compromise mitochondrial integrity [33], contributing to apoptosis initiation in MDR cells.

Consequently, the antioxidant response reflects cellular attempts to compensate redox imbalance. Upregulation of *GPX1* and *GSTP* in both cell lines (U87 and U87-TxR), together with selective induction of *GSR*, *SOD2*, *GPX4*, *TRX1,* and *TRXR1* mRNAs in U87-TxR, indicates activation of both GSH- and Trx-dependent defenses. The induction is more pronounced in the MDR cells, consistent with higher oxidative stress and an active but insufficient compensatory response that leaves U87-TxR cells vulnerable to ROS-mediated death.

The Trx–ASK1 axis links redox changes to apoptosis [31]. Increased TrxR1 protein (confirmed by immunostaining), together with elevated ASK1 protein in U87-TxR cells, supports ROS-driven release/activation of ASK1-dependent apoptotic signaling [34]. The absence of direct TrxR enzymatic inhibition by **2** and **5** suggests that TrxR1 upregulation is a response to oxidative pressure rather than a consequence of direct enzymatic blockade.

In the absence of rescue experiments using ROS scavengers, we therefore consider ROS as a key mediator rather than the only determinant of the cytotoxicity of adamantane–sclareol hybrids, recognizing the possible contribution of parallel pathways.

Our results suggest two possible mechanisms for the increase in ROS caused by adamantane–sclareol hybrids: (1) H_2_O_2_/peroxynitrite can oxidize Keap1 cysteines, stabilizing Nrf2 and inducing ARE-driven genes such as GPX1 and GSTP [35]. The observed induction of GPX1/GSTP aligns with this pathway. (2) Increased ASK1 protein levels and changes in the Trx system indicate activation of the Trx/ASK1 axis. Accordingly, the activation of multiple MAPKs can influence AP-1 and other transcription factors that control antioxidant and stress genes [34]. However, whether any of these mechanisms are activated remains to be clarified experimentally.

The combined observations of higher peroxide/peroxynitrite induction, compromised mitochondrial potential, and an overwhelmed antioxidant response rationalize why MDR U87-TxR cells display collateral sensitivity. MDR selection can impose metabolic and antioxidant liabilities; the adamantane–sclareol hybrids exploit that liability to trigger selective apoptosis in resistant cells. In our recent study, we investigated hybrids **2** and **5** against non-small cell lung carcinoma-sensitive and MDR P-gp-overexpressing cells [23]. We identified hybrid **2** as a P-gp inhibitor, while **5** was determined to be a P-gp substrate. The differences in collateral sensitivity observed between the two sensitive/MDR cancer cell models (glioblastoma and non-small cell lung carcinoma) for hybrid **5** could be attributed to cell type variations. In contrast, hybrid **2** exhibited strong collateral sensitivity in both models, suggesting a possible association with P-gp expression. However, to further elucidate this interaction, in silico docking studies are warranted.

Both hybrids increase intracellular accumulation of the P-gp substrate Rhodamine 123 in U87-TxR cells, indicating P-gp inhibition. The concentration- and time-dependent profiles differ: **5** shows concentration-dependent short-term inhibition that decreased with extended exposure, whereas **2** produces sustained inhibition up to 72 h. These differences align with combination data: **2** more consistently lowers the IC_50_ for PTX and shows stronger synergism with PTX across tested concentrations. Thus, the hybrids act through a dual mechanism—direct redox-mediated cytotoxicity in MDR cells plus functional inhibition of P-gp that restores sensitivity to chemotherapeutics [36].

The adamantane–sclareol hybrids **2** and **5** differ only by the heteroatom in a five-membered heteroaromatic ring (thiophene in **2**; furan in **5**). They have identical labdane cores and adamantyl-octyl amine substituents. The superior selectivity, sustained P-gp inhibition, and stronger PTX synergism of **2** suggest the thiphene-moiety-containing heterocycle confers favorable physicochemical or target-interaction properties (e.g., membrane affinity, nanoparticle behavior, or direct interaction with transporter/regulatory proteins) [37]. Indeed, our recent work showed that **2** has better nanoparticle characteristics with a narrower size distribution and a higher zeta potential than **5** [23].

Beyond their shared biological profile, the structural architectures of **2** and **5** suggest that each fragment contributes a distinct function to the overall activity spectrum. The sclareol backbone provides a diterpenoid framework with inherent antitumor properties and brain penetration potential [19], thereby serving as a privileged scaffold that is compatible with glioblastoma targeting and mitochondria-associated stress [20]. The added polymethylene diamine fragment, along with the attached thiophene (**2**) or furan (**5**) rings, act as a linker and an adjustable spacer that controls intermolecular packing and the surface charge of the resulting nanoparticles; the thiophene variant, in particular, is associated with more favorable colloidal properties and coincides with stronger MDR reversal by hybrid **2**. The adamantane substructure introduces a bulky, rigid, and highly lipophilic motif that is well suited to interact with lipid bilayers and membrane proteins [38], which is consistent with the observed P-gp modulation and supports the notion that this subunit enhances contact with the efflux transporter and cellular membranes. In this way, the hybrids can be viewed as modular constructs in which the sclareol core confers redox-active cytotoxicity, the linker adjusts nano- and interfacial behavior, and the adamantane moiety reinforces membrane and P-gp engagement, together yielding the combination of collateral sensitivity, ROS remodeling, and efflux inhibition detected in U87-TxR cells.

To clarify how hybrids inhibit P-gp and interact with redox systems, future work should include investigations of the P-gp ATPase activity, downstream responses to increased ROS such as induction of ARE-driven genes or ASK1 phosphorylation and downstream JNK/p38 activation assays after treatment with **2** and **5**, and experiments testing whether GPX4 loss in U87-TxR cells results from transcriptional repression or enhanced proteasome/autophagy inhibition.

## 4. Materials and Methods

### 4.1. Chemicals

The following chemicals and reagents were used in the experimental work: Minimal Essential Medium (MEM), Minimum Essential Medium Eagle (EMEM), Dulbecco’s Modified Eagle Medium (DMEM), DHR, Rho 123, TMRE, CCCP, DTNB or Ellman’s reagent, ATM (Sigma-Aldrich, Darmstadt, Germany); Dimethyl sulfoxide (DMSO); bovine serum albumin (BSA) (SERVA Electrophoresis GmbH, Heidelberg, Germany); DHE, TRIzol^®^ Reagent, MTT, High-Capacity cDNA Reverse Transcription Kit with RNase Inhibitor (Thermo Fisher Scientific, Waltham, MA, USA); Annexin V-FITC Apoptosis Staining detection kit (Abcam, Cambridge, UK); Fetal bovine serum (FBS), a mixture of antibiotics penicillin–streptomycin, L-glutamine (Capricorn Scientific, Ebsdorfergrund, Germany); methanol (Zorka-Pharma d.o.o., Hrastnik, Slovenia).

### 4.2. Drugs

Adamantane–sclareol hybrids were diluted in DMSO and 20 mM aliquots were kept at −20 °C. TQ was diluted in DMSO, and 10 mM aliquots were kept at −20 °C. PTX was obtained from Sigma-Aldrich, Darmstadt, Germany and kept at −20 °C as 1 mM aliquots. CDDP was purchased from Ebewe pharma, Unterach, Austria and kept at +4 °C. Working dilutions were prepared in deionized water immediately before the treatments.

### 4.3. Cells and Cell Culture

The human glioblastoma cell lines U87 and A-172 and the normal glial cell line SVG p12 were purchased from American Type Culture Collection (ATCC, Manassas, VA, USA). The U87-TxR cell line was originally selected from the U87 cell line by continuous exposure to gradually increasing concentrations of PTX over nine months (doi: 10.1016/j.biopha.2011.04.015). U87 and U87-TxR cells were grown in MEM, supplemented with 10% FBS, 2 mM L-glutamine, and a 5000 U/mL penicillin and 5 mg/mL streptomycin mixture. A-172 cells were cultivated in DMEM, supplemented with 10% FBS, 2 mM L-glutamine, and a 5000 U/mL penicillin and 5 mg/mL streptomycin mixture. The normal human glial cell line was grown in EMEM with alpha modification, supplemented with 10% FBS, 2 mM L-glutamine, a 5000 U/mL penicillin and 5 mg/mL streptomycin mixture, and 1% non-essential amino acids. All cell lines were grown in 25 cm^2^ and 75 cm^2^ flasks (Sarsted, Nümbrecht, Germany) until 70–80% confluence was reached. Thereafter, cells were trypsinized, counted using Burker-Turk hemocytometer on an inverted microscope, and seeded in appropriate densities (16,000 cells/cm^2^) for further experimental setup or maintenance in culture.

### 4.4. MTT Assay

Cell viability was measured using the colorimetric MTT assay based on the reduction of MTT into formazan dye. Cell lines were seeded into 96-well cell culture plates (4000 cells/well) in appropriate medium. After overnight incubation, cells were treated with increasing concentrations of **1**, **2**, **3**, **4**, **5**, and **6** (0.5, 1, 2, 4, and 8 µM). After 72 h treatment, MTT solution (4 mg/mL) was added and plates were incubated at 37 °C with 5% CO_2_ for 3 h. After incubation, formazan crystals were dissolved using 100 µL DMSO, resulting in a purple color. The absorbance was measured at 570 nm with a reference wavelength of 690 nm using an automated microplate reader (MultiskanSky, Thermo Scientific, USA). The IC50 values were determined by non-linear regression analysis in GraphPad Prism 8.0.2. software (GraphPad Software, Boston, MA, USA).

### 4.5. Preliminary Efficacy Study in Animals

Male immunodeficient mice (Rag1^−/−)^, aged 4–8 weeks, were used for xenograft studies. A total of 1 × 10^6^ U87 cells were injected subcutaneously into the rear axillary region to establish solid tumors. Tumor size and body weight were monitored twice weekly, and animals were observed for signs of morbidity or discomfort. Maximum tolerated dose (MTD) testing for **5** was performed in 6- to 8-week-old male Rag1 mice using two animals per dose group. Compound 5 was administered i.p. at doses of 7 mg/kg, 12.5 mg/kg, 25 mg/kg, 50 mg/kg, 100 mg/kg, and 200 mg/kg. The carrier consisted of 10% (*v*/*v*) DMSO with 5% (*v*/*v*) Tween-80 and was diluted in 0.9% (*w*/*v*) NaCl. Animals were monitored for weight loss, changes in feeding or neurological signs, and any morbidity to identify the highest non-toxic dose. For efficacy studies, Rag1 mice were inoculated subcutaneously with 1 × 10^6^ cells (mean starting weight ≈ 20 g); animals were then randomized into treatment groups. The compound **5** group (*n* = 5) received 7 mg/kg i.p. (two injections during the study period). Comparator groups included carrier control (*n* = 5). Compound **5** was formulated in the vehicle described above. The study was terminated when tumors in untreated mice reached approximately 11% of body weight. At termination, animals were euthanized and tumors were excised. Tumor volumes and body weights were recorded throughout the experiment and used to assess efficacy and tolerability.

The handling and experimentation of the animals were conducted under a licensed protocol approved by the IACUC and Greek authorities (License no. 5542/228006) and in accordance with Greek laws (PD 56/2013 and Circular 2215/117550/2013) and the guidelines of the European Union (2013/63/EU).

### 4.6. Combination Effect Analysis

The combined effects of **2** and **5** with PTX were studied in U87-TxR cells by MTT assay as previously described. In simultaneous treatments that lasted 72 h, three concentrations of **2** and **5** (0.5, 1, and 2 µM) were combined with three concentrations of PTX (0.1 μM, 0.2 μM, and 0.5 μM). Drug combination effects were evaluated using the Chou–Talalay method, which calculates the combination index (CI) value for the degree of interaction between the drugs. This method allows quantitative assessment of synergism (CI < 1), additivity (CI close to 1), or antagonism (CI > 1) across a range of dose–effect relationships. Calcusyn^®^ version 1.1 software was used for data analysis (Biosoft, Cambridge UK). Three data points were used for each single drug in each designed experiment. The non-constant ratio combination was chosen to assess the effect of both drugs in combination. The results are presented in a fraction-affected CI graph.

### 4.7. Cell Death Detection

To evaluate the extent of cell death upon treatment with adamantane–sclareol hybrids, apoptotic, necrotic, and viable cell populations were assessed using dual staining with AV/PI. U87 and U87-TxR cells were seeded in 6-well plates at a density of 150,000 cells per well and incubated overnight. Subsequently, cells were treated with **2** and **5** (2 µM and 4 µM) for 48 h. At the end of the treatment period, cells were collected (both adherent and floating), washed, and incubated with AV/PI in binding buffer. Samples were analyzed using a CytoFLEX flow cytometer (Beckman Coulter, Indianapolis, IN, USA), with fluorescence signals detected at 525 nm (AV) and 585 nm (PI). At least 10,000 events were recorded per each condition. The percentages of viable (AV^−^ PI^−^), early apoptotic (AV^+^ PI^−^), late apoptotic (AV^+^ PI^+^), and necrotic (AV^−^ PI^+^) cells were calculated using CytExpert 2.4.0.28 software (Beckman Coulter, Indianapolis, IN, USA).

### 4.8. Reactive Oxygen Species Detection

The levels of ROS were measured in U87 and U87-TxR cells by flow cytometry. The production of superoxide anions was detected using DHE fluorescence dye, while production of H_2_O_2_ and peroxynitrite anions was detected using DHR fluorescence dye. Cells were seeded in 6-well plates (150,000 cell/well) and incubated overnight. The cells were then treated with compounds **2** or **5** (4 μM) for 24 h and 48 h. CDDP (10 μM) was used as a positive control. After the treatment period, the cells were stained with DHE or DHR for 30 min in the dark. Following incubation, cells were harvested with trypsinization and washed twice in cold phosphate buffer solution (PBS). Fluorescence was measured on a CytoFlex flow cytometer (Beckman Coulter, Indianapolis, IN, USA). Green fluorescence from the oxidized DHR dye was detected at 525 nm, while the red fluorescence from ethidium derived from DHE was detected at 585 nm. At least 10,000 events were assayed for each sample. The results were analyzed by CytExpert 2.4.0.28 software (Beckman Coulter, Indianapolis, IN, USA).

### 4.9. TMRE-Based Measurement of Mitochondrial Membrane Potential

The cell-permeable fluorescent dye TMRE was used to evaluate mitochondrial membrane potential. TMRE selectively accumulates in active mitochondria with intact membrane potential. In contrast, depolarized or dysfunctional mitochondria exhibit decreased membrane potential, resulting in reduced dye accumulation and fluorescence intensity. U87 and U87-TxR cells were seeded in 6-well plates at a density of 150,000 cells per well. After 24 h, cells were treated with **2** and **5** (4 µM) and incubated for an additional 24 h. After treatment, cells were trypsinized, resuspended in complete medium containing 500 nM TMRE, and incubated for 30 min at 37 °C in the dark. CCCP (10 µM) was used as a positive control for mitochondrial depolarization and added 30 min before staining. For each sample, at least 10,000 events were collected. TMRE was detected at 525 nm. The results were calculated using CytExpert 2.4.0.28 software (Beckman Coulter, Indianapolis, IN, USA).

### 4.10. RNA Extraction and Reverse Transcription Reaction

Prior to RNA isolation, U87 and U87-TxR cell were treated with 4 μM **2** and **5** for 24 h. TRIzol^®^ Reagent was used to isolate total RNA according to manufacturer’s instructions. RNA concentration was determined with NanoPhotometer N60 (Implen GmbH, Munchen, Germany). Quality was determined using electrophoresis on a 1.3% agarose gel. Reverse transcription reaction was carried out using High-Capacity cDNA Reverse Transcription Kit with RNase Inhibitor.

### 4.11. Quantitative Real-Time PCR

Quantitative RT-PCR (RT-qPCR) was performed to evaluate mRNA expression levels of *CAT*, *GSR*, *SOD2*, *GPX1*, *GPX4*, *GST*, *TRX1*, and T*RXR1*, with *ACTB* used as the reference gene [39,40,41,42,43,44,45,46,47,48,49,50,51]. Reactions were carried out using Maxima SYBR Green/ROX qPCR Master Mix (Thermo Fisher Scientific, Waltham, MA, USA) in the QuantStudio™ 3 Real-Time PCR System (Applied Biosystems by Thermo Fisher Scientific). Thermocycling conditions consisted of an initial incubation at 50 °C for 5 min, followed by denaturation at 95 °C for 10 min and then 40 cycles of amplification with denaturation at 95 °C for 15 s and annealing/extension at 60 °C for 60 s. Relative gene expression levels were calculated using the ΔΔCt method, normalizing target gene expression to *ACTB*. All reactions were performed in technical triplicates.

### 4.12. Protein Expression Analysis

Flow cytometry was used to assess TrxR1 and ASK1 protein expression in the U87 and U87-TxR cell lines. Cells were seeded and allowed to attach overnight before exposure to compounds **2** and **5** at final concentrations of 2 μM and 4 μM. After 48 h treatment, cells were collected by trypsinization, rinsed with PBS, and subsequently fixed in 4% PFA for 10 min at room temperature. For permeabilization, ice-cold absolute methanol was added, and the samples were maintained at −20 °C overnight. Following this step, cells were washed in PBS and incubated for 1 h in 0.5% BSA/PBS to block nonspecific binding. Pelleted cells were then resuspended in primary antibodies diluted in 0.5% BSA/PBS [(ASK1, 1:200 (ab45178, Abcam, Cambridge, UK); TrxR1, 1:500 (ab124954, Abcam, Cambridge, UK)] and incubated overnight at +4 °C. Cells were then washed in PBS and incubated for 1 h at room temperature in the dark with fluorescently labeled anti-rabbit IgG secondary antibody (Alexa Fluor 488, ab150097, Abcam, Cambridge, UK), diluted 1:1000 in 0.5% BSA/PBS. Following incubation, samples were washed, resuspended in cold PBS, and analyzed by flow cytometry. A minimum of 10,000 events per sample were acquired, and fluorescence was detected in a green fluorescence channel (525 nm). Data acquisition and processing were performed using CytExpert software (version 2.4.0.28; Beckman Coulter, Indianapolis, IN, USA).

### 4.13. Colorimetric Detection of Cell Lysate TrxR Activity

TrxR enzyme activity in cell lysate was determined by the reduction of the TrxR substrate DTNB to the yellow product TNB in the presence of NADPH (Acros Organics, Thermo Fisher Scientific, Waltham, MA, USA). Lysate from human neuroblastoma SH-SY5Y cells was used for the TrxR assay. SH-SY5Y cells were grown to 80% confluence, washed twice with PBS, and detached using Hanks-based enzyme-free dissociation solution (Millipore, Billeri-ca, MA, USA). Collected cells were centrifuged at 2000 rpm for 3 min and rinsed in PBS buffer. After additional centrifugation, cells were lysed on ice using Lysis Buffer (50 mM Tris, 150 mM NaCl, 1 mM EDTA, 1% NP40, 0.1% SDS, 0.5% Na-deoxycholate, pH 7.8) containing protease inhibitor mix G (SERVA Electrophoresis GmbH, Heidelberg, Germany) and phosphatase inhibitor cocktail 3 (Sigma-Aldrich, Darmstadt, Germany). The cell lysates were centrifuged at 16,000× *g* for 15 min at 4 °C. The protein concentrations of the supernatants were determined using a Pierce^TM^ BCA protein assay kit (Thermo Fisher Scientific, Waltham, MA, USA). The reactions were run in 96-well plates, in a final volume of 100 μL, in 50 mM potassium phosphate buffer (pH 7.0) containing 50 μg of cell lysate proteins, 1 mM EDTA, 50 mM KCl, 0.2 mg/mL BSA, and 0.25 mM NADPH. The reaction mixture was incubated in a 90 μL reaction volume for 15 min at room temperature, with compounds **2** and **5** tested at a concentration of 50 µM. After incubation, 10 μL of DTNB was added to final concentration of 0.5 mM. Enzyme kinetics were monitored on an automated microplate reader (MultiskanSky, Thermo Fisher Scientific, Waltham, MA, USA) by measuring the increase in absorbance at 412 nm for 20 min. ATM (Sigma-Aldrich, Darmstadt, Germany), a potent TrxR inhibitor, was used as a positive control for TrxR inhibition, while DMSO was used as a negative control.

### 4.14. Rhodamine 123 Accumulation Assay

Flow cytometry was used to evaluate accumulation of fluorescent P-gp substrate Rho123. Studies were carried out with **2**, **5,** and **TQ** in U87-TxR cells with P-gp overexpression. Sensitive U87 cells were used as a positive control for Rho 123 accumulation. U87-TxR cells were seeded in 6-well plates at a density of 100,000 cells/well and incubated overnight. Then, the cells were treated with 1 μM and 2 μM of compounds **2** and **5** for 24 h, 48 h, and 72 h. Thereafter, cells were incubated with 2 μM Rho 123 for an additional 30 min at 37 °C in 5% CO_2_. In another experimental setting, 2 μM Rho123 was simultaneously applied with increasing concentrations of **2** or **5** (1 μM, 2 μM, and 4 μM) for 30 min. At the end of the accumulation period, the samples were washed twice, resuspended in 1 mL of cold PBS, and analyzed using a CytoFLEX flow cytometer (Beckman Coulter, Indianapolis, IN, USA). The fluorescence was read in a green fluorescence channel (525 nm). At least 10, 000 events were assayed for each sample. Data were analyzed using CytExpert 2.4.0.28 software (Beckman Coulter, Indianapolis, IN, USA).

### 4.15. Statistical Analysis

All experiments were performed in triplicate (*n* = 3). Data were analyzed using GraphPad Prism 8.0.2 (GraphPad Software, Boston, MA, USA). Cell death induction, ROS analysis (DHR and DHE), mitochondrial membrane potential analysis (TMRE), and time-dependent intracellular Rho123 accumulation analyses were assessed by two-way ANOVA followed by Dunnett’s multiple comparisons test, comparing each treatment group to the mean of the untreated control samples, while for the preliminary animal study, two-way ANOVA followed by Sidak’s multiple comparisons test was used. Concentration-dependent intracellular Rho123 accumulation and protein expression analyses were determined by one-way ANOVA followed by Dunnett’s multiple comparisons test, comparing each treatment group to the mean of the untreated control samples. For the quantitative Real-Time PCR statistical analysis was performed using two-way ANOVA followed by Sidak’s multiple comparisons test. For the MTT assay involving combined treatments, IC50 values were calculated using non-linear regression analysis in GraphPad Prism 8.0.2. Statistical analysis for combined effects was performed by two-way ANOVA, comparing combination treatments to PTX alone. The confidence interval was 95%.

## 5. Conclusions

Adamantane–sclareol hybrids **2** and **5** selectively target and kill P-gp-overexpressing U87-TxR cells by inducing oxidative stress through H_2_O_2_ and peroxynitrite. This process collapses the mitochondrial membrane potential and inhibits P-gp, thus sensitizing MDR glioblastoma cells to PTX. The MDR phenotype exhibits an asymmetric antioxidant profile (increased *GPX1* and *CAT* levels, decreased *GPX4*), which creates a vulnerability to lipid-ROS that these hybrids can exploit. Future research should confirm the role of lipid peroxides and establish causal links with ferroptosis, further elucidate the mechanisms of P-gp modulation, and expand studies on in vivo pharmacokinetics, toxicity, and orthotopic efficacy. The hybrids evaluated here fall within that conceptual space by combining a labdane (sclareol) core with adamantane to generate nanoparticles that modulate ROS and P-gp in MDR cells. The pending patent “Labdane diterpenes as a material for nanoparticles and their use”, therefore, provides a legal framework for protecting the broader platform of labdane-based nanoparticle materials and their therapeutic uses.

## 6. Patents

LABDANE DITERPENES AS A MATERIAL FOR NANOPARTICLES AND THEIR USE pending to The Intellectual Property Office of the Republic of Serbia (P-2025/0632).

## Figures and Tables

**Table 1:** Cytotoxicity of novel adamantane–sclareol hybrids in U87, U87-TxR, SVG p12, and A-172 cell lines.

IC50 (µM)						
Compound	U87	U87-TxR	Relative Resistance Factor	SVG p12	A-172	Selectivity Index
**1**	2.395 ± 0.061	2.081 ± 0.037	0.869	5.328 ± 0.132	2.472 ± 0.036	2.300 **^S^**
**2**	2.594 ± 0.068	1.964 ± 0.049	0.757 **^CS^**	6.197 ± 0.145	2.051 ± 0.370	2.813 **^S^**
**3**	2.201 ± 0.052	2.309 ± 0.052	1.049	9.29 ± 0.169	3.362 ± 0.057	3.541 **^S^**
**4**	1.254 ± 0.036	1.455 ± 0.027	1.160	0.918 ± 0.022	1.380 ± 0.018	0.686
**5**	5.133 ± 0.139	3.779 ± 0.118	0.736 **^CS^**	6.687 ± 0.128	4.735 ± 0.129	1.470
**6**	2.474 ± 0.066	1.907 ± 0.046	0.771 **^CS^**	1.163 ± 0.027	1.999 ± 0.026	0.547

**^CS^** Collateral sensitivity: The IC50 value for the indicated compound is lower by at least 20% in the MDR U87-TxR cells compared to the IC50 value obtained in the corresponding sensitive U87 cell line (relative resistance factor < 0.8). **^S^** Selectivity towards cancer cells: The IC50 value for a given compound in normal glial cell line SVG p12 is at least two-fold higher than the average IC50 value determined in the sensitive glioblastoma cells A-172 and U87 and the MDR glioblastoma U87-TxR cells.

**Table 2:** Relative reversal of PTX resistance by adamantane–sclareol hybrids in MDR glioblastoma cells.

Compounds	Conc. (µM)	IC50 _PTX_ (µM)	Relative Reversal
**PTX**		0.688 ± 0.022	
**2**	0.5	0.391 ± 0.013	1.75
	1.0	0.410 ± 0.011	1.68
	2.0	0.225 ± 0.008	3.05
**5**	0.5	0.797 ± 0.031	0.86
	1.0	0.371 ± 0.015	1.85
	2.0	0.390 ± 0.014	1.76

## Data Availability

The raw data supporting the conclusions of this article will be made available by the authors on request.

## Supplementary Materials

The following supporting information can be downloaded at: https://www.mdpi.com/article/10.3390/molecules30244756/s1, Figure S1: Antitumor activity and tolerability of compound 5 (PAS 114) in xenograft-bearing Rag1 mice. Figure S2: Cell death induction by adamantane–sclareol hybrids with Annexin V/PI staining in U87; Figure S3: Cell death induction by adamantane–sclareol hybrids with AV/PI staining in U87-TxR cells; Figure S4: Changes in ROS production in U87 and U87-TxR cells following treatment with **2** and **5**; Figure S5: Difference in the mRNA expression of antioxidant-related genes between U87 and U87-TxR cell lines; Figure S6: TrxR1 and ASK1 protein expression in U87 and U87-TxR cell lines; Figure S7: Curve showing changes in absorbance of DTNB, measured at a wavelength of 412 nm over 20 min; Figure S8: Flow cytometry profiles of rhodamine 123 accumulation upon treatment with adamantane–sclareol derivatives in U87-TxR cells, which overexpress P-glycoprotein.

## Abbreviations

The following abbreviations are used in this manuscript:

ABC	ATP-binding cassette
ARE	Antioxidant Response Element
ASK1	Apoptosis signal-regulating kinase 1
ATM	Aurothiomalate
AV/PI	Annexin V/Propidium Iodide
BSA	Bovine serum albumin
CAT	Catalase
CCCP	Carbonyl cyanide m-chlorophenyl hydrazone
CDDP	Cisplatin
CI	Combination index
CS	Collateral sensitivity
DMEM	Dulbecco’s Modified Eagle Medium
DHE	Dihydroethidium
DHR	Dihydrorhodamine 123
DMSO	Dimethyl sulfoxide
DTNB	5,5ʹ-Dithiobis(2-nitrobenzoic acid)
EMEM	Minimum Essential Medium Eagle
FBS	Fetal bovine serum
GPxs	Glutathione peroxidases
GSTs	Glutathione S-transferases
GSH	Glutathione
GSSG	Oxidized glutathione
JNK	c-Jun N-terminal kinase
Keap1	Kelch-like ECH-associated protein 1
MAPK	Mitogen-Activated Protein Kinase
MEM	Minimal Essential Medium
MDR	Multidrug resistance
MTT	3-[4,5-Dimethylthiazol-2-yl]-2,5-diphenyltetrazolium bromide
NADPH	Nicotinamide adenine dinucleotide phosphate
Nrf	Nuclear factor erythroid 2–related factor
PBS	Phosphate-buffered saline
P-gp	P-glycoprotein
PTX	Paclitaxel
Rho 123	Rhodamine 123
ROS	Reactive oxygen species
S	Selectivity toward glioblastoma cells
SI	Selectivity index
SOD	Superoxide dismutase
TMRE	Tetramethylrhodamine ethyl ester
TNB	5-Thio-2-nitrobenzoic acid
TQ	Tariquidar
Trx	Thioredoxin
TrxR	Thioredoxin reductase
