# Supplementary material for: Novel Adamantane–Sclareol Hybrids Exploit ROS Vulnerability to Overcome Multidrug-Resistance in Glioblastoma Cells
Source: Molecules. 2025 Dec 12;30(24):4756. doi: 10.3390/molecules30244756 (PMC12735718; doi:10.3390/molecules30244756)
Supplement: Supplementary file 1 [file molecules-30-04756-s001.zip › molecules-4030340-supplementary.pdf]

# Supplementary Material

# Novel Adamantane–Sclareol Hybrids Exploit ROS Vulnerability to Overcome Multidrug-Resistance in Glioblastoma Cells

**Ema Lupšić [1], Pavle Stojković [2], Marija Grozdanić [1], Nataša Terzić-Jovanović [3], Milica Pajović [1], Fani Koutsougianni [4], Dimitra Alexopoulou [4], Igor M. Opsenica [2], Milica Pešić [1*], Ana Podolski-Renić [1*]**

[1] Institute for Biological Research "Siniša Stanković" – National Institute of the Republic of Serbia, University of Belgrade, Despota Stefana 142, 11108 Belgrade, Serbia

[2] University of Belgrade – Faculty of Chemistry, PO Box 51, Studentski Trg 16, 11158 Belgrade, Serbia

[3] University of Belgrade - Institute of Chemistry, Technology, and Metallurgy, National Institute of the Republic of Serbia, Njegoševa 12, 11000 Belgrade, the Republic of Serbia

[4] Department of Pharmacology, Faculty of Medicine, School of Health Sciences, University of Thessaly, Panepistimiou 3 (Biopolis), 41500 Larissa, Greece

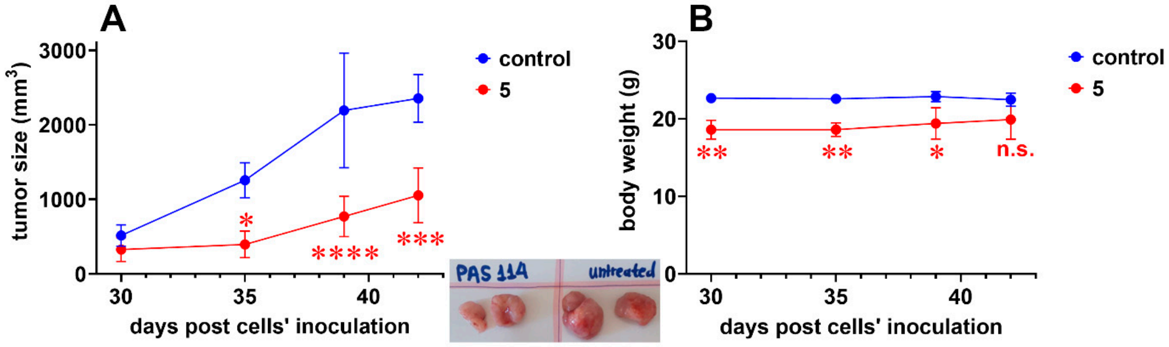


**Figure S1**. Antitumor activity and tolerability of compound **5** (PAS 114) in xenograft-bearing Rag1 mice. (A) Tumor growth curves showing tumor volume ($mm^3$) from day 30 to 42 after cell inoculation in untreated control with carrier (blue) and compound **5**–treated (red) groups (n=5). Time points reached statistical significance as indicated * $p < 0.05$; ** $p < 0.01$; *** $p < 0.001$. (B) Body weight of animals over the same period. Differences between groups * $p < 0.05$; ** $p < 0.01$; n.s. = non-significant. Photograph (center) shows representative excised tumors from **5** (PAS 114)–treated and untreated animals. Data are presented as mean ± SEM. Two-way ANOVA and a Sidak's multiple comparisons test.

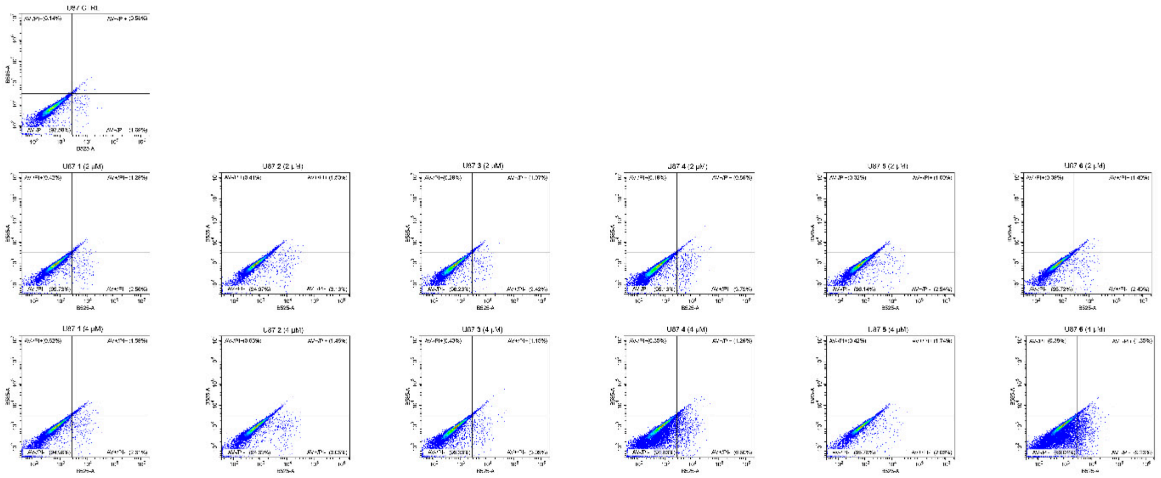

**Figure S2.** Cell death induction by adamantane-sclareol hybrids with AV/PI staining in U87 cells. Representative flow cytometry dot plots of U87 cells after treatment with six adamantane-hybrid compounds. Treatment lasted for 48 h. Staining allowed distinction between viable cells ($AV^-/PI^-$, lower left quadrant), early apoptotic cells ($AV^+/PI^-$, lower right quadrant), late apoptotic cells ($AV^+/PI^+$, upper right quadrant), and necrotic cells ($AV^-/PI^+$, upper left quadrant).

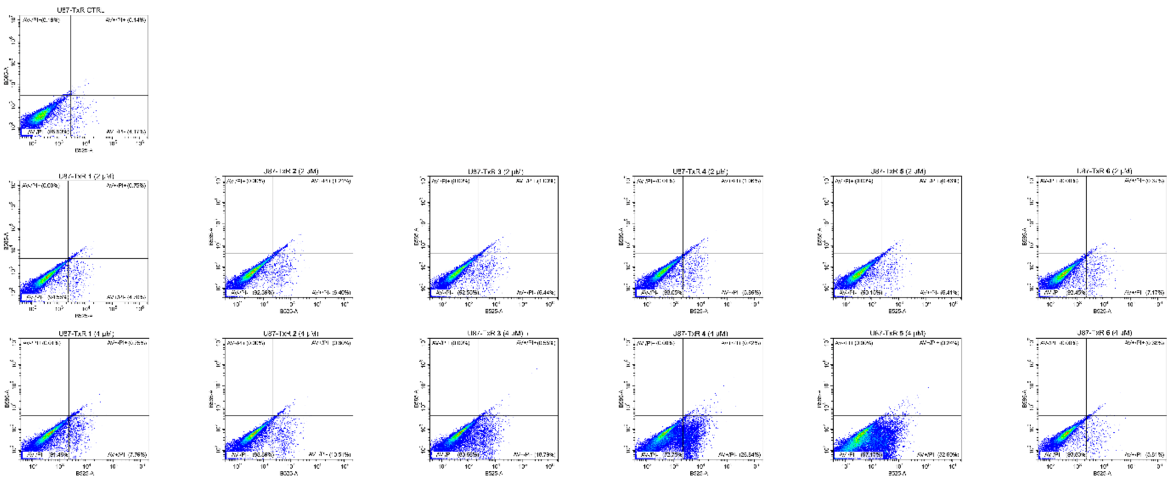

**Figure S3.** Cell death induction by adamantane-sclareol hybrids with AV/PI staining in U87-TxR cells. Representative flow cytometry dot plots of U87-TxR cells after treatment with six adamantane-hybrid compounds. Treatment lasted for 48 h. Staining allowed distinction between viable cells ($AV^-/PI^-$, lower left quadrant), early apoptotic cells ($AV^+/PI^-$, lower right quadrant), late apoptotic cells ($AV^+/PI^+$, upper right quadrant), and necrotic cells ($AV^-/PI^+$, upper left quadrant).

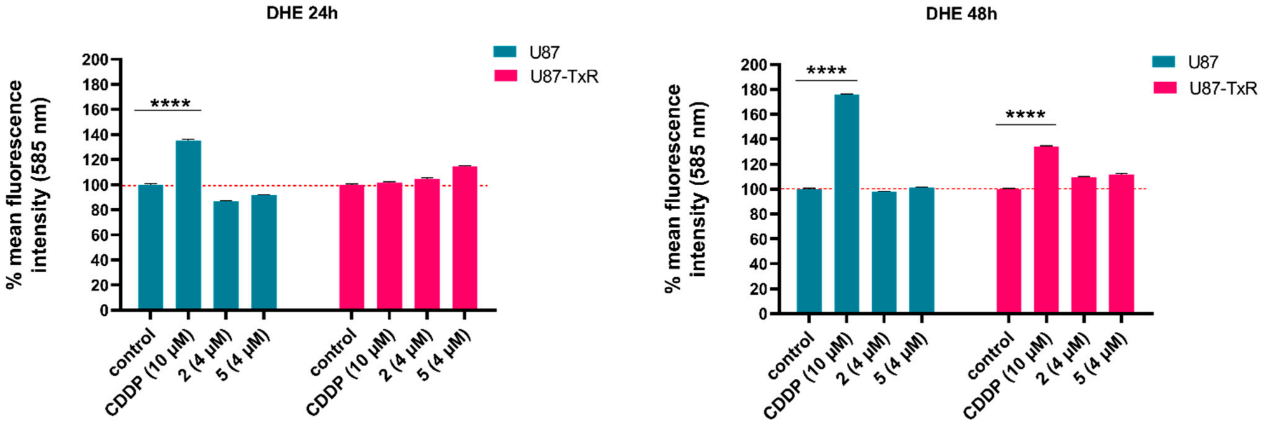


**Figure S4.** Changes in ROS production in U87 and U87-TxR cells following treatment with **2** and **5**. Changes in detection of superoxide anion levels by DHE after 24 h and 48 h treatment with **2** and **5**. Results are expressed as percentage of mean fluorescence intensity of untreated control. Three separate experiments were performed (n=3). Statistical analysis was performed using GraphPad Prism 8.0.2. with Two-way ANOVA and a Dunnett's multiple comparisons test. A statistically significant difference between the treated samples and the untreated control is indicated as ****p ≤ 0.0001.

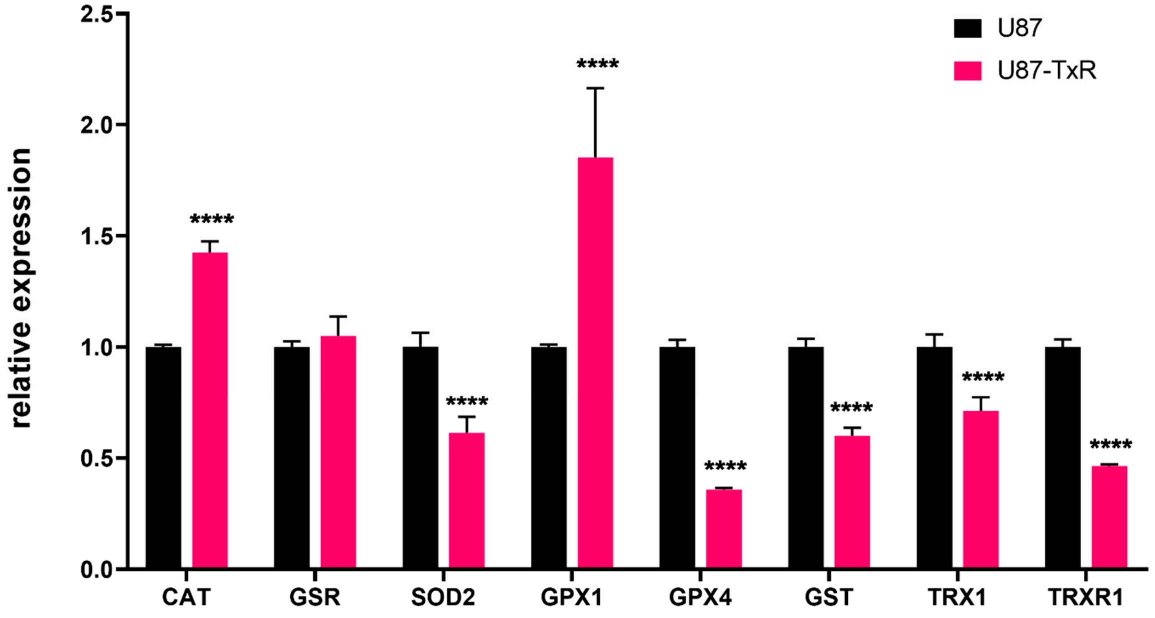


**Figure S5.** Difference in the mRNA expression of antioxidant-related genes between U87 and U87-TxR cell lines. Quantitative RT-PCR analysis of *CAT*, *GSR*, *SOD2*, *GPX1*, *GPX4*, *GSTP*, *TRX1*, and *TRXR1* mRNA expression in U87 and U87-TxR cells. Expression levels were normalized to the housekeeping gene ACTB and are presented relative to U87 cells. Data represents the mean ± SEM from three independent experiments. Statistical analysis was performed using Two-way ANOVA followed by Sidak's multiple comparisons test; statistically significant difference between the U87-TxR vs. U87: ****p ≤ 0.0001.

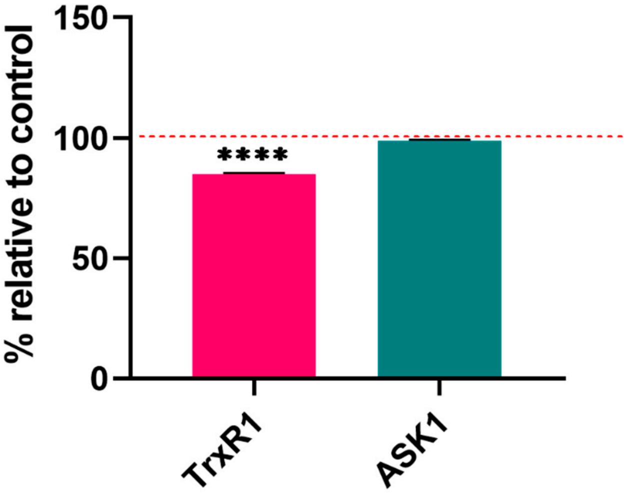


**Figure S6**. TrxR1 and ASK1 protein expression in U87 and U87-TxR cell lines. Data are presented as percentage relative to U87 cells (100%, red dashed line), with statistical significance to the U87 cells ****$p < 0.0001$. Statistical analysis was performed using One-way ANOVA followed by Dunnett's post hoc test.

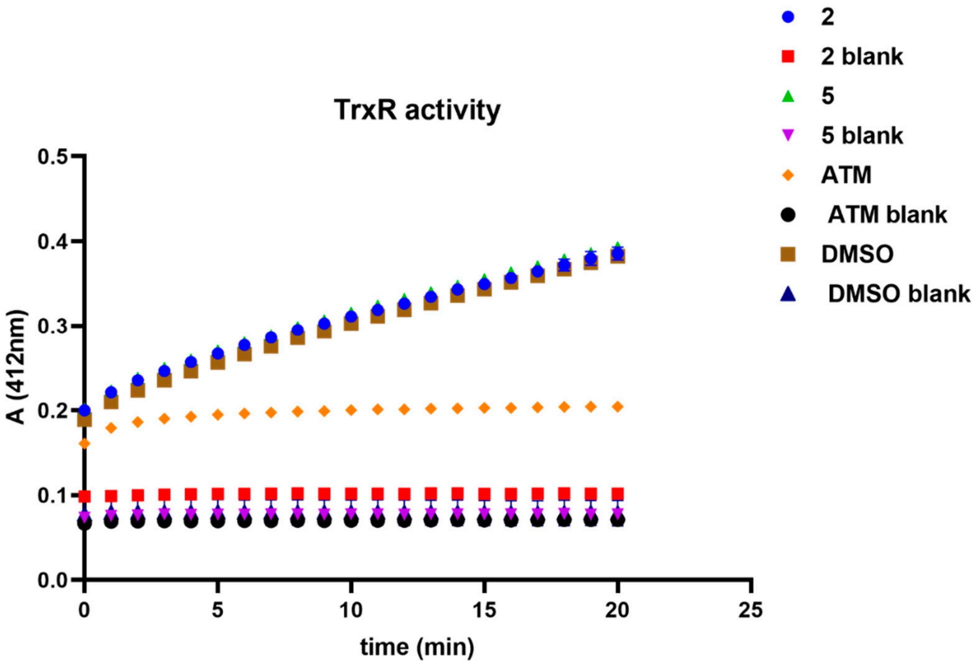


**Figure S7.** Curve showing changes in absorbance of DTNB, measured at a wavelength of 412 nm over 20 minutes. Absorbance changes of DTNB over time were measured in the presence of **2** (50 μM), **5** (50 μM), ATM (20 μM), and DMSO. Each reaction group was compared with absorbance values obtained in the absence of cell lysate protein (blank).

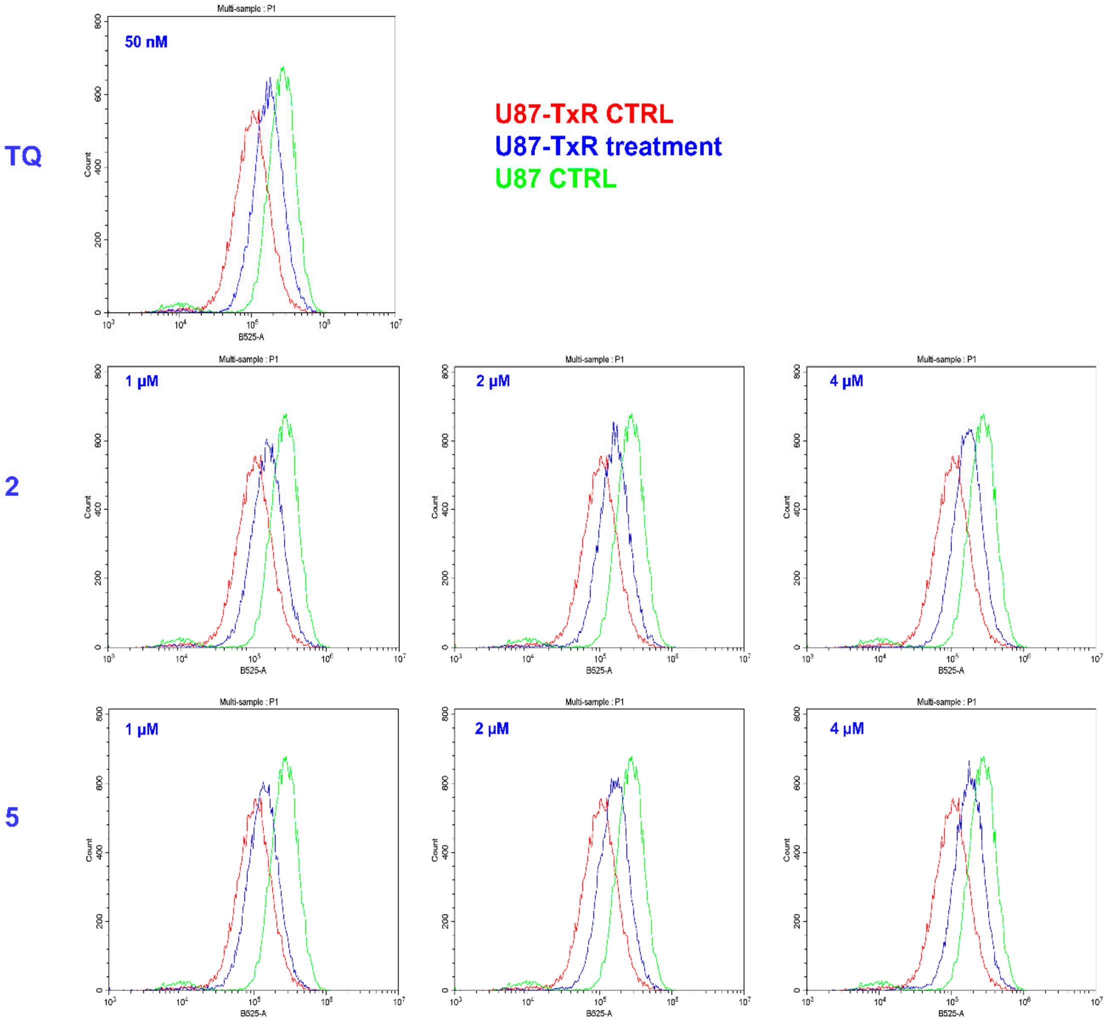


**Figure S8**. Flow cytometry profiles of rhodamine 123 accumulation upon treatment with adamantane-sclareol derivatives in U87-TxR cells, which overexpress P-glycoprotein. Sensitive U87 cells, which do not express P-glycoprotein, served as a positive control for rhodamine 123 accumulation. TQ (tariquidar) was applied as a positive control for P-glycoprotein inhibition. At least 10,000 events were recorded per sample. Shift in the fluorescence profile illustrate the effects of tested compounds.
